# Editorial: Heterogeneity and plasticity of prostate cancer

**DOI:** 10.3389/fmolb.2023.1228126

**Published:** 2023-06-14

**Authors:** Zoran Culig, Mohit Kumar Jolly, Karel Souček

**Affiliations:** ^1^ Department of Urology, Medical University of Innsbruck, Innsbruck, Austria; ^2^ Centre for BioSystems Science and Engineering, Indian Institute of Science, Bangalore, India; ^3^ Institute of Biophysics Academy of Science of the Czech Republic, Brno, Czech Republic; ^4^ Clinical Research Center, St. Anne’s University Hospital, Brno, Czech Republic

**Keywords:** prostate cancer, heterogeneity, plasticity, therapy resistance, transcription factors, oncogenic miRNA, tumor-suppressive miRNA, immunotherapy

Prostate cancer represents a highly heterogeneous and one of the most clinically common malignancies in men. Despite the improvement in the management of localized prostate cancer, therapy options for the advanced stages of the disease remain limited. Resistance to endocrine and chemotherapy treatments in prostate cancer is a subject of ongoing discussion and investigation. Limited understanding of the extent of cellular plasticity, clonality and intratumoral heterogeneity in the context of therapy resistance remains one of the main bottlenecks of current prostate cancer research. There is an increased number of novel drugs available. However, the mechanisms that are relevant to therapy resistance are incompletely understood and additional studies are needed. The ultimate goal may be to establish evidence-based drug sequences on the basis of understanding the action of transcription factors, oncogenic and tumor-suppressive miRNA in prostate cancer. Another challenge for experimental therapy is the existence of stem cells which may be targeted by some drugs originally implemented in non-malignant diseases.

In this Research Topic, we focus on promising, recent, and novel research trends in prostate cancer research. Despite the general improvement of cancer therapy efficacy, a benefit for prostate cancer patients especially in advanced stages is rather limited. Understanding of heterogeneity and plasticity of prostate cancer, its relation to the therapy and disease progression might identify novel strategies for effective treatment. Novel insights into the role of transcription factors and specific regulatory miRNA are particularly interesting.

Perturbed transcriptional control is a major driving mechanism in the development of prostate cancer (PCa). Deng et al. provided a comprehensive review summarizing the role of key epigenetic regulators in gene transcription and the progression of prostate cancer. The authors emphasized the impact of epigenetic modifications, such as histone or DNA methylation, histone acetylation, and non-coding RNA, on the activity of the androgen receptor (AR) and the expression of AR target genes. The review also discussed the diagnostic and therapeutic potential of epigenetic regulators in prostate cancer, particularly in combination with antiandrogen therapies (Deng et al.).

Current research efforts are focused on understanding the regulation of epithelial-to-mesenchymal transition (EMT) in the context of enzalutamide resistance. SNAIL has emerged as a key factor associated with increased JAK/STAT activity in this process (Ware et al.). SNAIL, a member of the transforming growth factor-β network, plays a critical role in tumor growth *in vivo*. The JAK/STAT pathway, known for its therapeutic potential, exhibits enhanced phosphorylation in the presence of the cytokine interleukin-6 (IL-6), which is upregulated during prostate cancer progression. IL-6 also promotes tumor metastasis, and other signaling pathways such as mitogen-activated protein kinase and Akt may become active following IL-6 treatment. Moreover, highly phosphorylated STAT3 has been observed in prostate cancer stem cells, highlighting the interconnectedness between signaling pathways and immunotherapy in prostate cancer. Anti-androgen therapy has been associated with increased expression of programmed cell death 1 ligand 1 (PD-L1) and elevated infiltration and activity of CD8^+^ T cells in prostate cancer (Sommer et al.).

PD-L1 is frequently expressed in immune and stromal cells in the prostate, suggesting its potential as a target for immunotherapies. In this context, the use of interleukin-15 (IL-15) in immunotherapy shows promise, as it can expand and activate CD8^+^ T cells and natural killer cells (Esteves et al.). Cytotopic modification of IL-15 has demonstrated efficacy in prostate tumors, further supporting immunotherapy as a viable treatment option. Collectively, these findings suggest a potential shift from conventional therapies to immunotherapy. Sipuleucel, for instance, could be used clinically to increase survival in asymptomatic patients with prostate cancer.

In a study conducted by Wang et al., the differential expression of pyroptosis-related genes associated with tumor immune infiltration was analyzed in prostate cancer. Pyroptosis, a form of cell death linked to inflammation, has been implicated in the progression of various tumors. The analysis identified eight genes that predict the risk of biochemical relapse in prostate cancer, providing potential prognostic biomarkers (Wang et al.).

The findings presented in studies on prostate cancer plasticity provide insights into the development of novel drugs that target immunosuppressive factors in cancer. Understanding the heterogeneity and plasticity of prostate cancer will pave the way for more effective therapeutic strategies. In summary, these studies collectively contribute to our understanding of the heterogeneity and plasticity of prostate cancer and offer potential avenues for personalized medicine, targeted therapies, and immunotherapy. Further research in this field is likely to yield novel approaches to overcome therapy resistance and improve patient outcomes in prostate cancer.

